# Sphingosine-1-Phosphate Receptor 2 Regulates Proinflammatory Cytokine Production and Osteoclastogenesis

**DOI:** 10.1371/journal.pone.0156303

**Published:** 2016-05-25

**Authors:** Hong Yu

**Affiliations:** Department of Oral Health Sciences, Center for Oral Health Research, Medical University of South Carolina, Charleston, South Carolina, United States of America; University of Pecs Medical School, HUNGARY

## Abstract

Sphingosine-1-phosphate receptor 2 (S1PR2) couples with the Gi, Gq, and G_12/13_ group of proteins, which modulate an array of cellular signaling pathways and affect immune responses to multiple stimuli. In this study, we demonstrated that knockdown of S1PR2 by a specific S1PR2 shRNA lentiviral vector significantly inhibited IL-1β, IL-6, and TNF-α protein levels induced by oral pathogen *Aggregatibacter actinomycetemcomitans* (*A*. *actinomycetemcomitans*) in murine bone marrow-derived monocytes and macrophages (BMMs) compared with controls. In addition, knockdown of S1PR2 by the S1PR2 shRNA lentiviral vector suppressed p-PI3K, p-ERK, p-JNK, p-p38, and p-NF-κBp65 protein expressions induced by *A*. *actinomycetemcomitans*. Furthermore, bone marrow cells treated with the S1PR2 shRNA lentiviral vector inhibited osteoclastogenesis induced by RANKL compared with controls. The S1PR2 shRNA suppressed the mRNA levels of six osteoclastogenic factors including nuclear factor of activated T-cells cytoplasmic calcineurin-dependent 1 (NFATc1), cathepsin K (Ctsk), acid phosphatase 5 (Acp5), osteoclast-associated receptor (Oscar), dendritic cells specific transmembrane protein (Dcstamp), and osteoclast stimulatory transmembrane protein (Ocstamp) in bone marrow cells. We conclude that S1PR2 plays an essential role in modulating proinflammatory cytokine production and osteoclastogenesis. Blocking S1PR2 signaling might be a novel therapeutic strategy to treat inflammatory bone loss diseases.

## Introduction

Sphingosine-1-phosphate (S1P) is a bioactive sphingolipid, which can be generated in most mammalian cells by various stimuli [1, 2]. Our previous study demonstrated that *Aggregatibacter actinomycetemcomitans* (*A*. *actinomycetemcomitans*), an oral pathogen associated with localized aggressive periodontitis, stimulated the generation of S1P in RAW 264.7 cells, a murine macrophage cell line [3]. Additionally, we demonstrated that reduction in generation of S1P in sphingosine kinase 1-deficient mice attenuated periodontal inflammation and reduced alveolar bone loss induced by *A*. *actinomycetemcomitans* compared with wild type mice [4]. Although it is known that oral pathogens cause inflammatory bone loss, the cell signaling pathways that regulate the inflammatory bone loss response have not been completely elucidated.

Monocytes and macrophages are major sources of proinflammatory cytokines in chronic inflammatory diseases. During inflammatory pathogenesis, bacterial pathogens activate various cellular signaling cascades including phosphoinositide 3-kinase (PI3K), mitogen-activated protein kinases (MAPKs), and nuclear factor kappa-B (NF-κB) pathways. The MAPKs include the extracellular signal-regulated kinase (ERK), c-Jun N-terminal kinase (JNK), and p38 MAPK. Activation of these signaling pathways results in proinflammatory cytokine release. Additionally, monocytes and macrophages are osteoclast precursors, which can fuse to form multinucleated mature osteoclasts [5]. Osteoclastogenesis is regulated by cytokines including M-CSF/CSF1, RANKL, and osteoprotegerin (OPG), which are key factors for osteoclastogenesis [6–8]. M-CSF (CSF1), generated by osteoblasts and bone marrow stromal cells, binds with its receptor CSF1R on osteoclast precursors, supporting their survival and proliferation [8]. RANKL, generated mainly by osteoblasts, mesenchymal stem cells, and T cells, binds with its receptor RANK on osteoclast precursors, promoting the differentiation of osteoclasts [6, 7]. OPG, produced mainly by bone marrow stromal cells, B lymphocytes, and dendritic cells, functions as a decoy receptor of RANKL [6, 7]. In addition, proinflammatory cytokines, such as IL-1 and TNF-α, also enhance osteoclastogenesis [6–8]. During osteoclast differentiation, Nfatc1 is considered the master transcription factor [9]. Nfatc1 regulates transcription of many osteoclastogenic genes, including Ctsk, Acp5, Oscar, Dcstamp, and Ocstamp [10–13].

S1P binds with five G protein-coupled cell surface S1P receptors (S1PR1-S1PR5), initiating various signaling pathways [1, 14]. One of the five S1PRs, S1PR2, [also called endothelial differentiation G-protein coupled receptor 5 (EDG5), AGR16, or H218] presents in many tissues and cell types including BMMs and fibroblasts [15–17]. S1PR2 couples with the Gi, Gq, and G_12/13_ group of proteins [15, 18], which modulate an array of cellular signaling pathways and affect many immune responses, including responses to bacterial lipopolysaccharide (LPS) [17], bile acid [19], histamine [20], vitamin D [21], and insulin [22, 23]. Previous studies demonstrate that S1PR2 regulates vascular inflammation and atherosclerosis [17]. *Apoe*^-/-^S1pr2^-/-^ mice showed greatly attenuated atherosclerosis compared with *Apoe*^-/-^S1pr2^+/+^ mice [17]. Additionally, S1PR2 inhibits the migration of monocytes and macrophages (osteoclast precursors) [16]. S1PR2-deficient mice exhibit moderate osteopetrosis [16]. Treating wild type mice with S1PR2 antagonist JTE013 alleviated osteoporosis induced by RANKL [16]. These studies demonstrated the important role of S1PR2 in regulating inflammatory response and bone homeostasis. However, the role of S1PR2 in inflammatory bone loss response is not elucidated. Previously, we showed that treating murine BMMs with FTY720, a multiple S1PRs modulator [24, 25], reduced IL-1β, IL-6, and TNF-α production induced by *A*. *actinomycetemcomitans* [26]. In addition, FTY720 suppressed osteoclastogenesis in BMMs induced by RANKL with or without *A*. *actinomycetemcomitans* stimulation [26]. Our study suggested that S1PRs might regulate proinflammatory cytokine production and osteoclastogenesis. However, it is unclear which S1PRs play a major role in modulating the proinflammatory cytokine production and osteoclastogenesis. In this study, we used the small hairpin RNA (shRNA) technique to knockdown S1PR2 gene expression and determine the role of S1PR2 in inflammatory cytokine release and osteoclastogenesis.

## Materials and Methods

### Animals and bone marrow-derived monocytes and macrophages (BMMs)

All experimental protocols were approved by the Institutional Animal Care and Use Committee at the Medical University of South Carolina. The animal study was performed in accordance with ARRIVE guidelines for animal research. Six to eight-week-old male C57BL/6J mice were purchased from Jackson Laboratory (Bar Harbor, ME). Bone marrow (BM) cells were harvested from mice by flushing BM with complete minimal essential media (MEM)-α (Life Technologies, Grand Island, NY, USA), supplemented with 10% fetal bovine serum (FBS), 100 U/mL penicillin, and 100 μg/mL streptomycin. To separate BMMs from stromal cells, BM cells were plated in 10 cm cell culture dishes and incubated at 37°C with 5% CO_2_ overnight. The suspended BM progenitor cells were transferred to a new cell culture dish and cultured for seven days in complete MEM-α media supplemented with 50 ng/mL recombinant murine M-CSF (R& D systems, Minneapolis, MN, USA) to allow cells to differentiate into BMMs.

### Generation of shRNA lentivirus

Murine S1PR2 MISSION shRNA plasmid DNA (TRCN0000028917), MISSION pLKO.1-puro non-mammalian shRNA control plasimd DNA (SHC002), and Human Embryonic Kidney (HEK) 293 cells were obtained from the ShRNA Shared Technology Resource at Medical University of South Carolina. To generate lentiviral shRNA vectors, HEK293 cells were co-transfected with S1PR2 shRNA plasmid DNA/ or control shRNA plasmid DNA along with lentiviral packaging plasmids pCMV-VSV-G and pCMV-dR8.2 dvpr (Addgene, Cambridge, MA, USA) using lipofectamine 2000 (Life Technologies). The supernatant was collected at 72 h after transfection and ultracentrifuged at 25,000 rpm for 1.5 h at 4°C using a Beckman Ultracentrifuge (Beckman Coulter, Indianapolis, IN, USA). The viral pellet was resuspended in serum-free DMEM medium, and viral titer was determined by a HIV-1 p24 Antigen ELISA kit (Zeptometrix, Buffalo, NY, USA).

### Culture of *A*. *actinomycetemcomitans*

*A*. *actinomycetemcomitans* (ATCC 43718) was purchased from American Type Culture Collection (Manassas, VA, USA). Bacterial colonies were grown on Difco^TM^ brain heart infusion agar plates (BD Biosciences, Sparks, MD, USA) and cultured in Bacto^TM^ brain heart infusion broth (BD Biosciences) for 24 h at 37°C with 10% CO_2_. Bacteria were centrifuged, washed with PBS with 5% glycerol, and resuspended in PBS with 5% glycerol. Bacterial concentration was determined by measuring bacterial optical density and by bacterial plating on brain heart infusion agar plates (OD_600_ = 1, about 3x 10^7^ colony forming unit, CFU/mL). *A*. *actinomycetemcomitans* stimulated cell culture media (*Aa*-media) was obtained by filter-sterilization of cell culture media derived from BMMs cultured with *A*. *actinomycetemcomitans* (1.5 CFU/cell) for 24 h.

### Enzyme-linked immunosorbent assay (ELISA)

IL-1β in cell lysate, IL-6 and TNF-α protein levels in cell culture media of BMMs were quantified by ELISA kits (R& D Systems). The concentration of cytokines was normalized by protein concentration, determined by a DC protein Assay Kit (Bio-Rad Laboratories, Hercules, CA, USA) in cell lysate.

### Western Blot analysis

Protein was extracted from BMMs by RIPA cell lysis buffer (Cell signaling Technology, Danvers, MA, USA). Total protein (30 μg) was loaded on 10% Tris-HCl gels, electro-transferred to nitrocellulose membranes, blocked, and incubated overnight at 4°C with primary antibody. The antibody to S1PR2 (TA314890) was obtained from OriGene Technologies (Rockville, MD, USA). The antibodies to p-PI3K (4228), p-ERK (4377), p-JNK (4668), p-p38 (4511), p-NF-κB p65 (3033), and GAPDH (2118) were purchased from Cell Signaling Technology (Danvers, MA, USA). After washing, the nitrocellulose membranes were incubated at room temperature for 1 h with horseradish peroxidase-conjugated secondary antibodies (Cell Signaling Technology) and developed using SuperSignal West Pico Chemiluminescent Substrate (Life Technologies Grand Island, NY, USA). Digital images and protein densitometry were analyzed by a G-BOX chemiluminescence imaging system (Syngene, Frederick, MD, USA).

### Osteoclastogenesis assay and tartrate-resistant acid phosphatase (TRAP) staining

Murine BM cells were cultured for three days in complete MEM-α media supplemented with 50 ng/mL recombinant murine M-CSF to allow BM progenitor cells to proliferate and differentiate. The BM cells were plated in new culture dishes and BM cells were either untreated or treated with lentivirus containing either S1PR2 shRNA or control shRNA (moi 20). After lentiviral infection for 5 h, the cells were cultured in complete MEM-α media containing both M-CSF (50 ng/ml, R&D systems) and RANKL (100 ng/ml, R&D Systems). A control group of cells were cultured with only M-CSF. The cell culture media was change at 48 h and 72 h after lentiviral infection. After lentiviral infection for 72 h, the cells were treated with or without *Aa*-stimulated media (100ul/ml) for 24 h. TRAP staining was performed in cultured BM cells using a leukocyte acid phosphatase kit (Sigma Aldrich, St. Louis, MO, USA). Pictures were taken using a Nikon Eclipse TS-100 inverted microscope. Image analysis was performed using Visiopharm 5.0 software (Visiopharm, Hoersholm, Denmark).

### Bone resorption assay

Murine BM cells were cultured for three days in complete MEM-α media supplemented with 50 ng/mL recombinant murine M-CSF and plated in a calcium phosphate-coated 48-well plate (Cosmo Bio USA, Carlsbad, CA). Cells were either untreated or treated with lentivirus containing either S1PR2 shRNA or control shRNA (moi 20). After lentiviral infection for 5 h, the cells were cultured in complete MEM-α media containing both M-CSF (50 ng/ml) and RANKL (500 ng/ml). A control group of cells were cultured with only M-CSF. On the 3^rd^ and 5^th^ day after lentiviral treatment, the cell culture media was changed with or without RANKL, and cells were either untreated or treated with *Aa*-stimulated media (100μL/well). Seven days after treatment, cells were removed by treatment with 5% sodium hypochlorite for 5 min. After washing and drying the plate, bone resorption pit images were taken by a Nikon Eclipse TS-100 inverted microscope and analyzed by Visiopharm 5.0 software.

### RNA extraction and real time PCR

Total RNA was isolated from cells using TRIZOL (Life Technologies) according to the manufacturer’s instructions. Complementary DNA was synthesized by a TaqMan reverse transcription kit (Life Technologies) using 1000 ng of total RNA. Real time PCR was performed using a StepOnePlus Real-Time PCR System (Life Technologies). PCR condition was used as follows: 50°C for 2 min, 95°C for 10 min, and 40 cycles of 95°C for 15 s, 60°C for 1 min. The following amplicon primers were obtained from Life Technologies: CSF1 (Mm00432686_m1), CSF1R (Mm01266652_m1), RANKL (Mm00441906_m1), RANK (Mm00437132_m1), OPG (Mm01205928_m1), NFATc1 (Mm00479445_m1), Ctsk (Mm00484039_m1), Acp5 (Mm00475698_m1), Oscar (Mm00558665_m1), Dcstamp (Mm04209236_m1), Ocstamp (Mm00512445_m1), S1PR1 (Mm02619656_s1), S1PR3 (Mm02620181_s1), S1PR4 (Mm00468695_s1), S1PR5 (Mm02620565_s1), and GAPDH (Mm99999915_g1). The mouse S1PR2 primers (PrimePCR^TM^ SYBR® Green Assay) were obtained from Bio-Rad Laboratories (Hercules, CA, USA). Amplicon concentration was determined using threshold cycle values compared with standard curves for each primer. Sample mRNA levels were normalized to an endogenous control GAPDH expression and expressed as fold changes compared with control groups.

### Statistical Analysis

All experiments were performed in triplicate with BM cells from mice. Data were analyzed by one-way ANOVA with Holm-Sidak’s multiple comparisons test. All statistical tests were performed using GraphPad Prism software (GraphPad Software Inc., La Jolla CA, USA). Values are expressed as mean ± standard error of the mean (SEM). A *P* value of 0.05 or less was considered significant.

## Results

### Knockdown of S1PR2 inhibited IL-1β, IL-6, and TNF-α protein production induced by *A*. *actinomycetemcomitans* in BMMs

**Fig 1:**
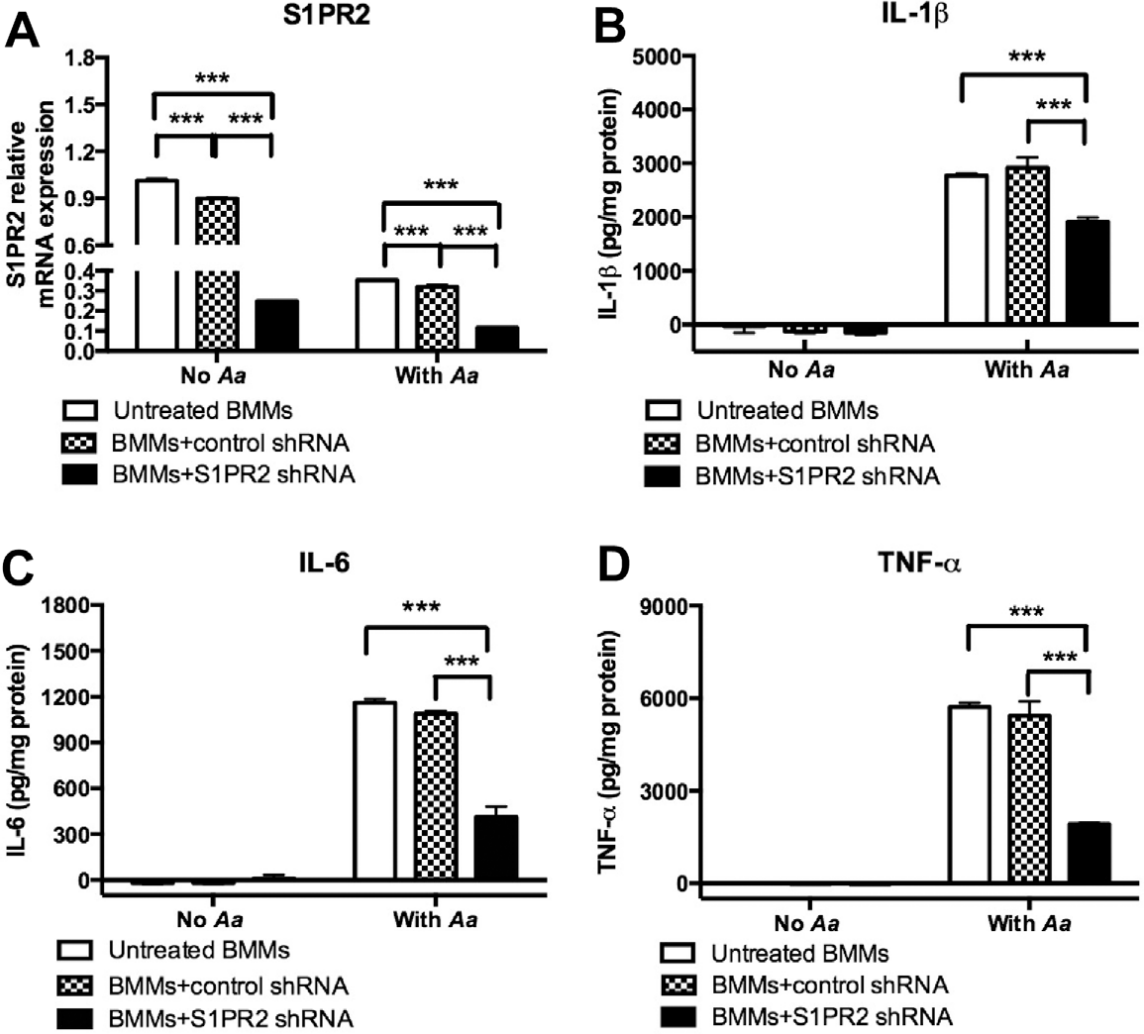
Knockdown of S1PR2 significantly decreased IL-1b, IL-6, and TNF-a protein expressions induced by *A*. *actinomycetemcomitans* (*Aa*) in BMMs. Murine BMMs were uninfected, infected with a S1PR2 shRNA lentivirus, or infected with a control shRNA lentivirus (moi 50) for 72 h. Then the cells were either unstimulated or stimulated with *Aa* (1.5 CFU/cell) for 4 h (S1PR2 mRNA assay) or 8 h (cytokine protein assays). (A) S1PR2 mRNA expression was determined by real time PCR, (B) IL-1β, (C) IL-6, and (D) TNF-α protein levels were quantified by ELISA and normalized by protein levels in cell lysates. The data are representatives from three separate experiments (n = 3, ***P<0.001).

To determine the role of S1PR2 in proinflammatory cytokine production induced by bacterial pathogen, BMMs derived from C57BL/6J mice were untreated, treated with a S1PR2 shRNA lentiviral vector, or treated with a control shRNA lentiviral vector (moi 50). Then, BMMs were either un-stimulated or stimulated with *A*. *actinomycetemcomitans* (1.5 CFU/cell). As shown in Fig 1A, S1PR2 shRNA significantly decreased S1PR2 mRNA expression by 72.3% in BMMs without bacterial stimulation and by 63.5% in BMMs 4 h after bacterial stimulation as compared with the control shRNA treatment. The control shRNA also slightly reduced S1PR2 mRNA by 11.2% before bacterial stimulation and by 9.3% after bacterial stimulation compared with the S1PR2 mRNA levels in cells without lentiviral infection. The S1PR2 shRNA did not significantly decrease S1PR1, S1PR3, S1PR4, and S1PR5 mRNA levels compared with control treatments (data not shown). Neither lentiviral vector induced a significant cytokine response in BMMs without bacterial stimulation (Fig 1B–1D). In contrast, S1PR2 shRNA significantly reduced IL-1β, IL-6, and TNF-α protein expressions induced by *A*. *actinomycetemcomitans* compared with controls (Fig 1B–1D). S1PR2 shRNA significantly decreased IL-1β by 30.4%, IL-6 by 62.2%, and TNF-α by 64.7% in BMMs induced by *A*. *actinomycetemcomitans* compared with the control shRNA group. In summary, S1PR2 shRNA specifically decreased S1PR2 mRNA expression, which subsequently suppressed IL-1β, IL-6, and TNF-α protein generation induced by the oral pathogen *A*. *actinomycetemcomitans*.

### Knockdown of S1PR2 attenuated S1PR2, p-PI3K, p-ERK, p-JNK, p-p38, and p-NF-kBp65 protein expressions in BMMs

**Fig 2:**
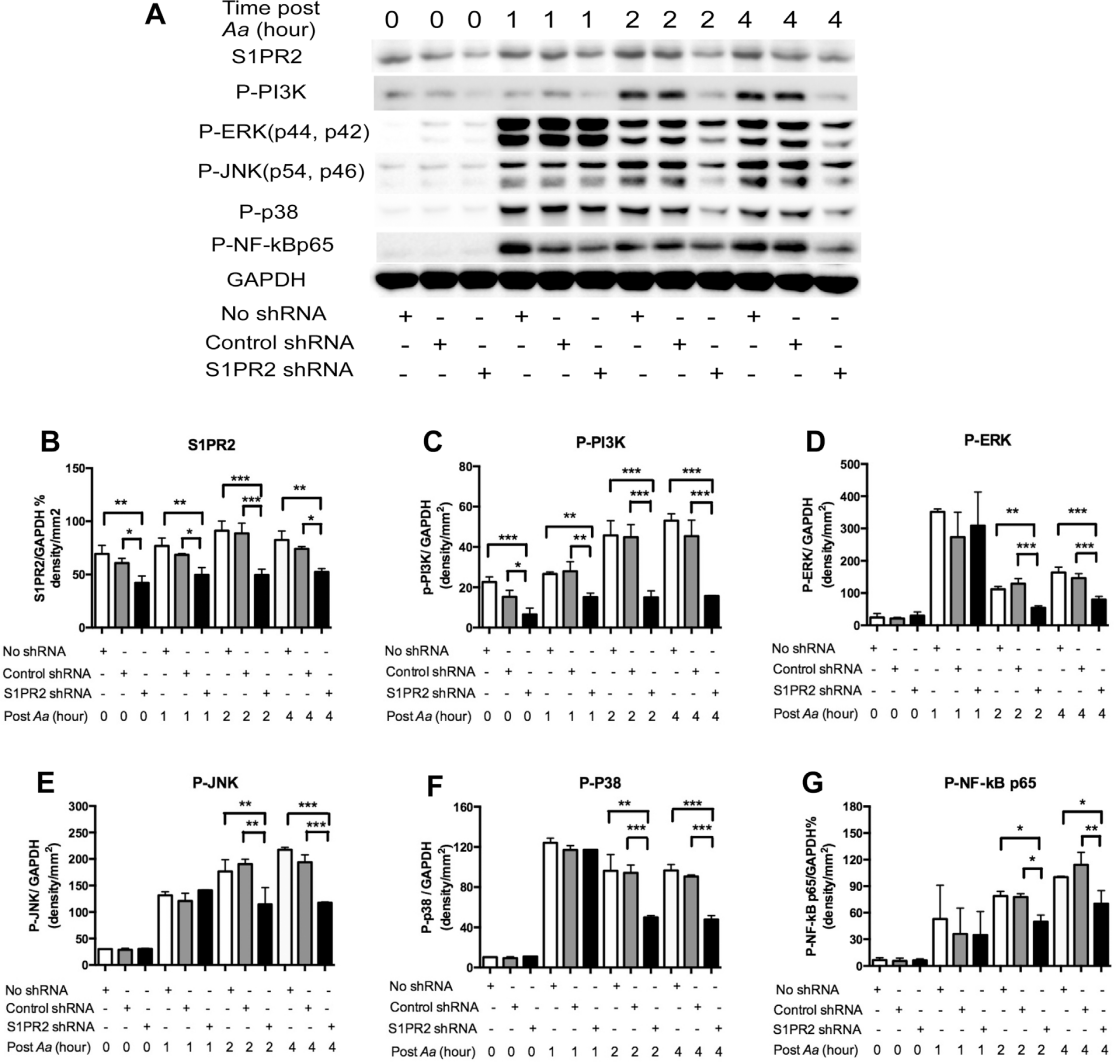
Knockdown of S1PR2 attenuated S1PR2, p-PI3K, p-ERK, p-JNK, p-p38, and p-NF-kBp65 protein expressions in BMMs. Murine BMMs were uninfected, infected with a S1PR2 shRNA lentivirus, or infected with a control shRNA lentivirus (moi 50) for 72 h. Then the cells were either unstimulated or stimulated with *Aa* (1.5 CFU/cell) for 1 to 4 h. (A) S1PR2, p-PI3K, p-ERK, p-JNK, p-p38, and p-NF-κBp65 protein expressions were evaluated by Western Blot. (B) S1PR2 protein density, (C) p-PI3K protein density, (D) p-ERK protein density, (E) p-JNK protein density, (F) p-p38 protein density, and (G) p-NF-κBp65 protein density were analyzed and normalized by GAPDH protein expression. The data are representatives from three separate experiments (n = 3, *P<0.05, **P<0.01, ***P<0.001).

To further elucidate which signaling pathways were affected by S1PR2 in regulating the immune response induced by *A*. *actinomycetemcomitans*, we performed Western blot assays in BMMs either untreated with lentivirus, treated with the S1PR2 shRNA lentivirus, or treated with the control shRNA lentivirus (moi 50), with or without *A*. *actinomycetemcomitans* (1.5 CFU/cell) stimulation. As shown in Fig 2A and 2B, *A*. *actinomycetemcomitans* stimulation increased S1PR2 protein expression. In BMMs uninfected with lentivirus, S1PR2 protein levels increased by 10.9%, 31.5%, and 19.0%, respectively, at 1, 2, and 4 h post bacterial stimulation compared with the S1PR2 basal level in unstimulated cells. BMMs treated with S1PR2 shRNA exhibited significantly decreased S1PR2 and p-PI3K levels before and after bacterial stimulation (Fig 2A–2C). S1PR2 shRNA reduced S1PR2 protein by 30.6% before bacterial stimulation and by 27.3%, 44.1%, and 21.8%, respectively, at 1, 2, and 4 h after bacterial stimulation compared with the control shRNA group (Fig 2A and 2B). S1PR2 shRNA also decreased p-PI3K by 57.1% before bacterial stimulation and by 45.7%, 66.6% and 65.4%, respectively, at 1, 2, and 4 h after bacterial stimulation compared with the control shRNA group (Fig 2A–2C). In contrast, there were no significant differences of p-ERK, p-JNK, p-p38, and p-NF-κBp65 protein expressions between S1PR2 shRNA group and the control shRNA group before bacterial stimulation and 1 h after bacterial stimulation (Fig 2A and 2D–2G). However, we observed significant reductions of p-ERK, p-JNK, p-p38, and p-NF-κBp65 at 2 and 4 h after bacterial stimulation in BMMs treated with S1PR2 shRNA compared with cells treated with the control shRNA (Fig 2A and 2D–2G). These results support that knockdown of S1PR2 by the S1PR2 shRNA specifically attenuated S1PR2 protein and subsequently reduced p-PI3K, p-ERK, p-JNK, p-p38, and pNF-κBp65 signaling pathways, which could contribute to the down-regulation of the proinflammatory cytokine response stimulated by *A*. *actinomycetemcomitans*.

### Knockdown of S1PR2 suppressed osteoclastogenesis induced by RANKL with or without treatment with *Aa*-stimulated media

**Fig 3:**
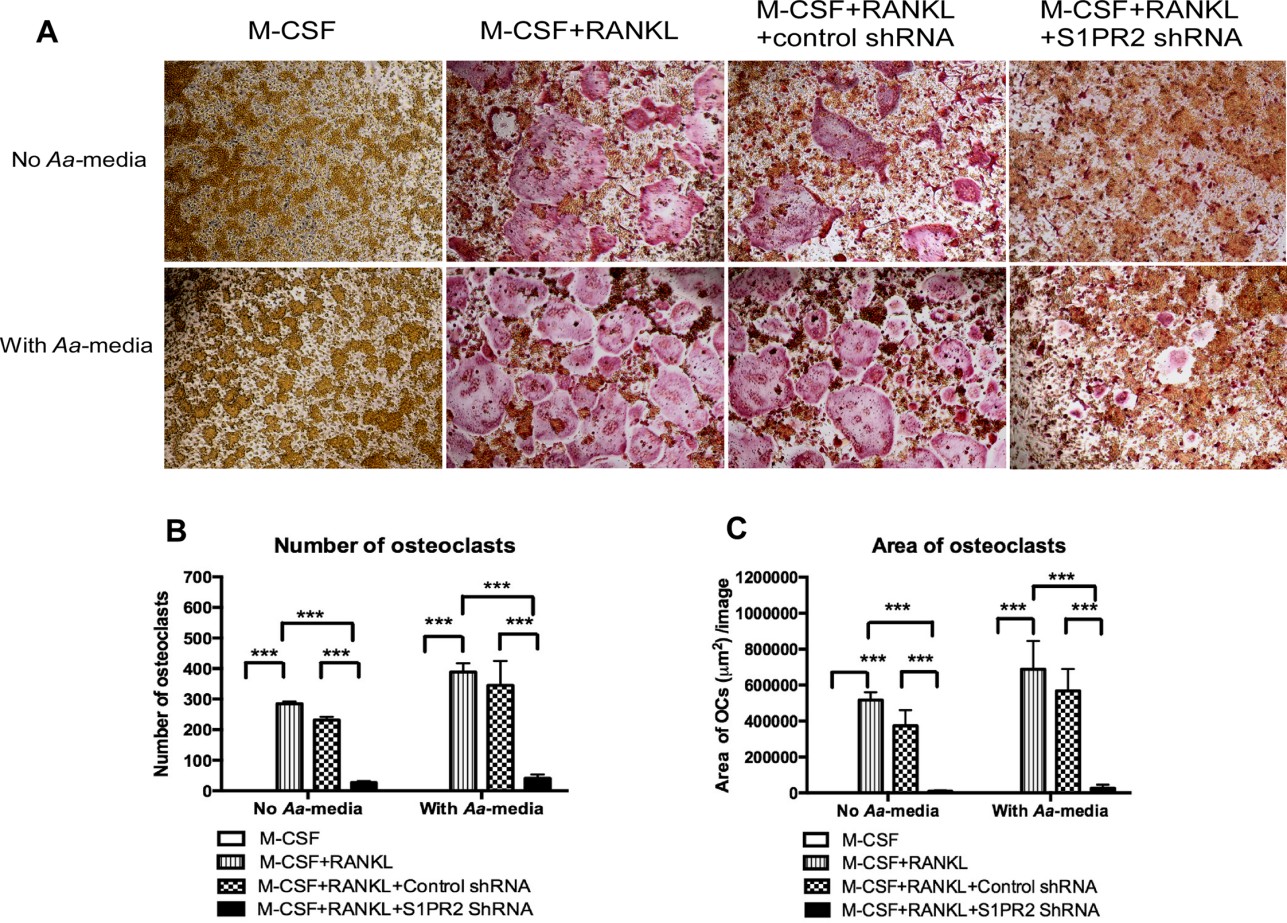
Knockdown of S1PR2 suppressed osteoclastogenesis in BM cells induced by RANKL. BM cells were uninfected, infected with a S1PR2 shRNA lentivirus, or infected with a control shRNA lentivirus (moi 20), and co-cultured with M-CSF and RANKL as described in Methods. A control group of cells were cultured only with M-CSF. BM cells were either untreated or treated with *A*. *actinomycetemcomitans*-stimulated media (*Aa*-media) for 24 h. (A) Representative images show TRAP-stained cells with and without *Aa*-media stimulation. Pictures were taken at 100x magnification. (B) Number of TRAP^+^ multinucleated (more than 3 nuclei) osteoclasts/well (96-well) and (C) Total areas of osteoclasts/image were quantified. The data are representatives from three separate experiments (n = 3, ***P<0.001).

Previous study showed that phosphoinositide signaling controls the activation of Nfatc1 and influences osteoclastogenesis [27]. Additionally, proinflammatory cytokines promote the differentiation of osteoclasts [28]. Since knockdown of S1PR2 by S1PR2 shRNA significantly inhibited PI3K signaling and attenuated IL-1β, IL-6, and TNF-α proinflammatory cytokine release induced by *A*. *actinomycetemcomitans*, we hypothesized that knockdown of S1PR2 could suppress osteoclastogenesis. To test our hypothesis, murine BM cells were untreated, treated with the S1PR2 shRNA lentivirus or the control shRNA lentivirus (moi 20). Cells were cultured with both M-CSF (50 ng/mL) and RANKL (100 ng/mL). A control group of BM cells were not infected with lentivirus and treated with M-CSF (50 ng/mL) alone. After lentiviral infection for 72 h, the cells were either unstimulated or stimulated with *Aa*-media for 24 h. As shown in Fig 3A, there were no TRAP^+^ osteoclasts in cells treated with only M-CSF, with or without *Aa*-media stimulation. There were many TRAP^+^ multinucleated osteoclasts in cells either uninfected with lentivirus or treated with the control shRNA lentivirus in the presence of both M-CSF and RANKL with or without *Aa*-media stimulation. In contrast, S1PR2 shRNA treatment decreased both size and number of TRAP^+^ multinucleated osteoclasts compared with control groups (Fig 3A). Quantification of the number of osteoclasts (Fig 3B) showed that S1PR2 shRNA decreased the number of osteoclasts by 88.6% compared with the control shRNA in cells without *Aa*-media stimulation. *Aa*-media stimulation increased the number the osteoclasts 1.4-fold. In addition, S1PR2 shRNA decreased the number of osteoclasts by 88.1% compared with the control shRNA group after *Aa*-media stimulation. The control shRNA also slightly reduced the number of osteoclasts by 18.9% in cells without *Aa*-media stimulation and by 11.2% in cells with *Aa*-media stimulation compared with cells without lentivirus treatment. However, there was no statistically significant difference between the control shRNA group and the uninfected group. Quantification of total area of osteoclasts per image revealed that S1PR2 shRNA reduced the area of osteoclasts by 97.4% in BM cells without *Aa*-media stimulation and by 95.3% in BM cells with *Aa*-media stimulation compared with the control shRNA group (Fig 3C). The control shRNA slightly decreased the area of osteoclasts by 27.8% in BM cells without *Aa*-media stimulation and by 17.5% in BM cells with *Aa*-media stimulation compared with BM cells uninfected with lentivirus. However, statistically there was no significant difference between the control shRNA group and uninfected group.

**Fig 4:**
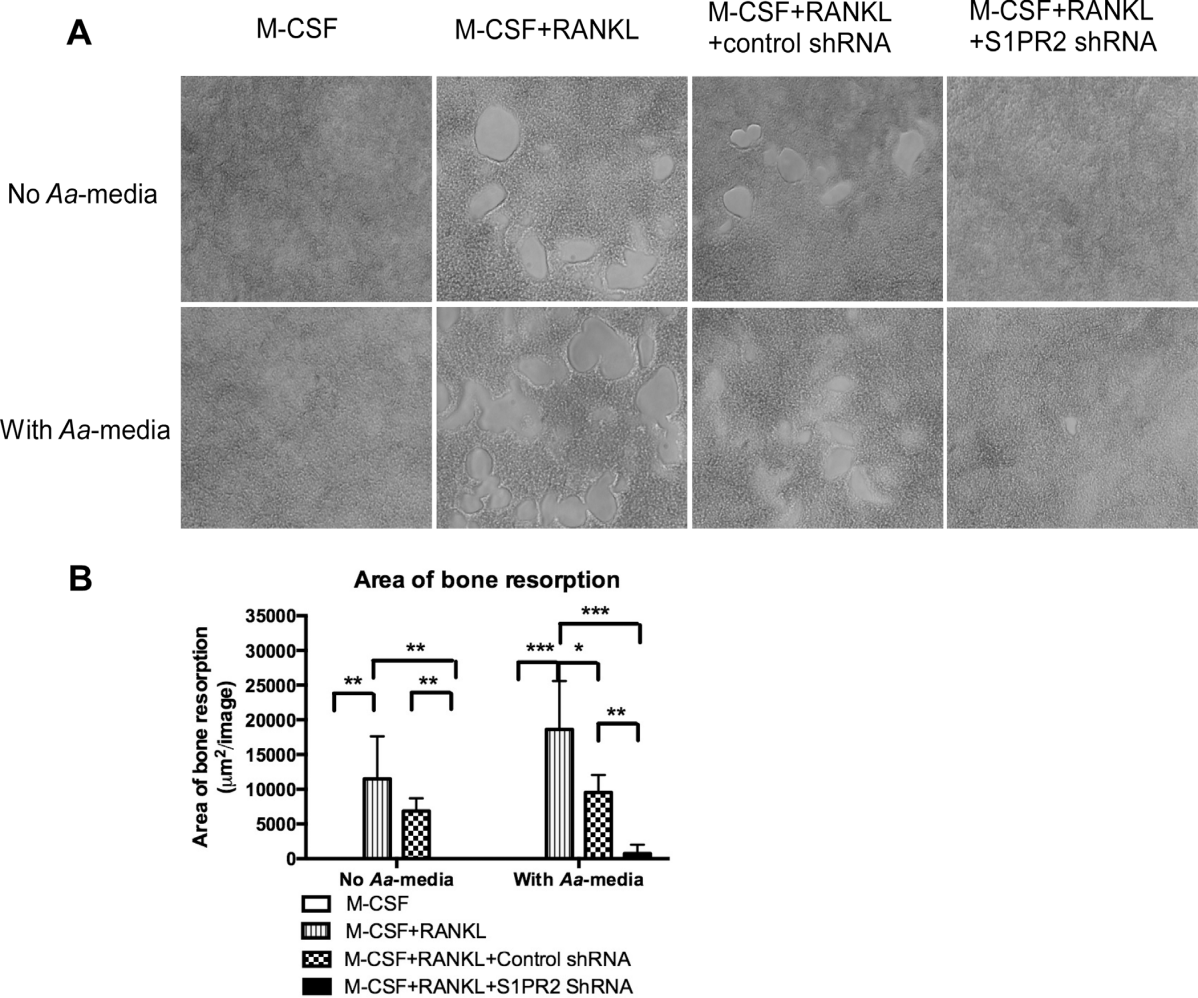
Knockdown of S1PR2 inhibited bone resorption in BM cells induced by RANKL. BM cells were uninfected, infected with a S1PR2 shRNA lentivirus, or infected with a control shRNA lentivirus (moi 20), and co-cultured with M-CSF and RANKL, with or without *A*. *actinomycetemcomitans*-stimulated media (*Aa*-media) treatment as described in Methods. Control groups of cells were cultured only with M-CSF with or without *Aa*-media treatment. (A) Representative images show bone resorption pits. Pictures were taken at 100x magnification. (B) Total areas of bone resorption pits /image were quantified. The data are representatives from three separate experiments (n = 3, *P<0.05, ** *P*<0.01, *** *P*<0.001).

To further define the role of S1PR2 in bone resorbing activity, a bone resorption assay was performed. Fig 4A showed that there was no bone resorption pits in cells treated with only M-CSF, with or without *Aa*-media stimulation. RANKL induced the formation of bone resorption pits in BM cells either uninfected with lentivirus or infected with the control shRNA lentivirus with or without *Aa*-media stimulation. In contrast, BM cells treated with the S1PR2 shRNA lentivirus significantly suppressed the formation of bone resorption pits induced by RANKL with or without *Aa*-media stimulation compared with controls. Quantification of total area of bone resorption pits per image revealed that S1PR2 shRNA decreased the area of bone resorption by 100% in BM cells without *Aa*-media stimulation and by 92.3% in BM cells with *Aa*-media stimulation compared with the control shRNA group (Fig 4B). The control shRNA also reduced the area of bone resorption by 40.3% in BM cells without *Aa*-media stimulation and by 48.7% in BM cells with *Aa*-media stimulation compared with BM cells uninfected with lentivirus.

### Knockdown of S1PR2 attenuated Nfatc1, Ctsk, Acp5, Oscar, Dcstamp, and Ocstamp mRNA expressions

**Fig 5:**
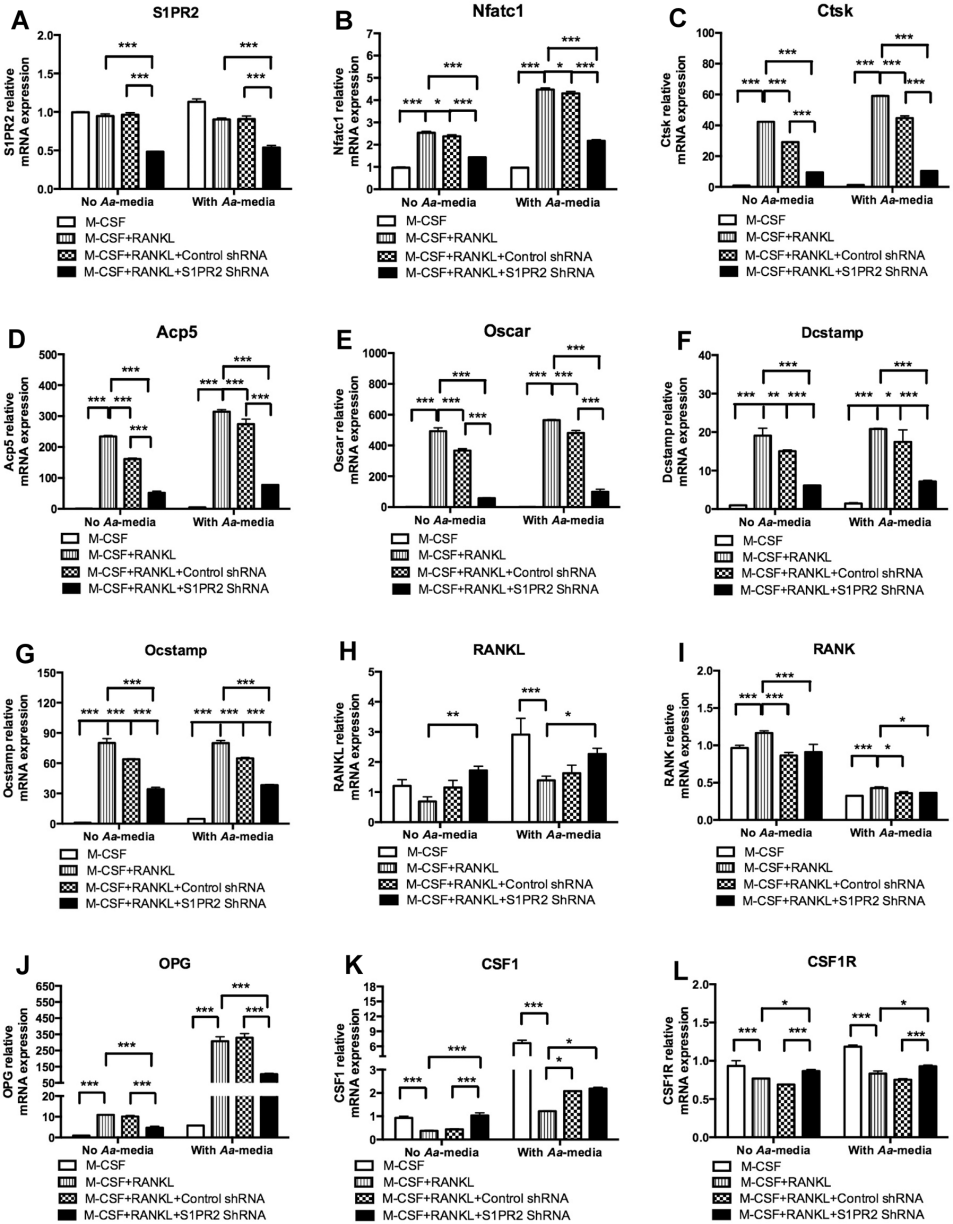
Knockdown of S1PR2 significantly attenuated Nfatc1, Ctsk, Acp5, Oscar, Dcstamp, and Ocstamp mRNA expressions in BM cells with or without treatment with *A*. *actinomycetemcomitans*-stimulated media (*Aa*-media). BM cells were uninfected, infected with a S1PR2 shRNA lentivirus, or infected with a control shRNA lentivirus (moi 20), and co-cultured with M-CSF and RANKL as described in Methods. A control group of cells were cultured only with M-CSF. BM cells were either unstimulated or stimulated with *Aa*- media for 4 h. (A) S1PR2 mRNA, (B) Nfatc1 mRNA, (C) Ctsk mRNA, (D) Acp5 mRNA, (E**)** Oscar mRNA, (F) Dcstamp mRNA, (G) Ocstamp mRNA, (H) RANKL mRNA, (I) RANK mRNA, (J) OPG mRNA, (K) CSF1 mRNA, and (L) CSF1R mRNA levels were quantified by real time PCR and normalized by GAPDH expression. The data are representatives from three separate experiments (n = 3, *P<0.05, ***P<0.001).

To elucidate the mechanisms associated with the role of S1PR2 on osteoclastogenesis, we analyzed the mRNA expressions of genes associated with osteoclastogenesis including Nfatc1, Ctsk, Acp5, Oscar, Dcstamp, Ocstamp, RANKL, OPG, RANK, CSF1, and CSF1R in BM cells untreated, treated with S1PR2 shRNA lentivirus, or treated with the control shRNA lentivirus (moi 20), with or without *Aa*-media stimulation. As shown in Fig 5A, S1PR2 shRNA decreased S1PR2 mRNA level by 49.7% in cells without *Aa*-media stimulation and decreased by 34.6% in cells 4 h after *Aa*-media stimulation as compared with the control shRNA treatment. As shown in Fig 5B–5G, S1PR2 shRNA significantly suppressed Nfatc1, Ctsk, Acp5, Oscar, Dcstamp, and Ocstamp mRNA levels in BM cells with or without *Aa*-media stimulation compared with these mRNA levels in cells treated with the control shRNA. S1PR2 shRNA reduced the mRNA levels of Nfatc1 by 40.0%, Ctsk by 67.6%, Acp5 by 67.9%, Oscar by 84.1%, Dcstamp by 59.4%, and Ocstamp by 46.6% in BM cells without *Aa*-media stimulation compared with these mRNA levels in cells treated with the control shRNA. In addition, S1PR2 shRNA reduced the mRNA levels of Nfatc1 by 49.4%, Ctsk by 76.8%, Acp5 by 71.9%, Oscar by 79.3%, Dcstamp by 57.2%, and Ocstamp by 40.9% in BM cells 4 h after *Aa*-media stimulation compared with these mRNA levels in BM cells treated with the control shRNA. However, we also observed that BM cells treated with the control shRNA also decreased the mRNA levels of Nfatc1 by 6.2%, Ctsk by 31.1%, Acp5 by 31.3%, Oscar by 25.5%, Dcstamp by 21.2%, and Ocstamp by 20.0% in cells without *Aa*-media stimulation, and reduced the mRNA levels of Nfatc1 by 3.9%, Ctsk by 24.2%, Acp5 by 12.7, Oscar by 14.7%, Dcstamp by 19.5%, and Ocstamp by 18.9% in cells stimulated with *Aa*-media compared with these mRNA levels in cells without lentiviral infection (Fig 5B–5G).

As shown in Fig 5H, *Aa*-media increased RANKL mRNA expression. However, cells treated with RANKL exhibited reduced RANKL mRNA expression compared with that in cells treated with only M-CSF. Both S1PR2 shRNA and the control shRNA slightly increased RANKL expression compared with RANKL mRNA level in uninfected cells treated with both M-CSF and RANKL. Compared with the control shRNA treatment, S1PR2 shRNA showed a trend of increasing RANKL mRNA level, but there was no significant difference between S1PR2 shRNA group and the control shRNA group. Both lentiviral treatments slightly decreased RANK mRNA expression (Fig 5I). RANK was reduced in cells treated with control shRNA, either unstimulated (a 25.9% reduction) or stimulated with *Aa*-media (a 15.8% reduction, Fig 5I). RANK was also reduced in cells treated with S1PR2 shRNA, either unstimulated (a 21.9% reduction) or stimulated with *Aa*-media (a 14.8% reduction, Fig 5I). There was no significant difference of RANK mRNA level between S1PR2 shRNA group and the control shRNA group (Fig 5I). S1PR2 shRNA reduced OPG by 53.2% and 70.8%, respectively, in cells either unstimulated or stimulated with *Aa*-media compared with the control shRNA treatment (Fig 5J). S1PR2 shRNA slightly increased CSF1 2.3-fold and 5.0%, respectively, in cells either unstimulated or stimulated with *Aa*-media compared with the control shRNA treatment (Fig 5K). S1PR2 shRNA increased CSF1R by 25.8% and 23.0%, respectively, in cells either unstimulated or stimulated with *Aa*-media compared with the control shRNA treatment (Fig 5L). In summary, Knockdown of S1PR2 by S1PR2 shRNA significantly attenuated Nfatc1, Ctsk, Acp5, Oscar, Dcstamp, and Ocstamp mRNA levels compared with the control shRNA group. There were no significant differences of RANKL and RANK mRNA levels between S1PR2 shRNA group and the control shRNA group. S1PR2 shRNA decreased OPG mRNA level, but it slightly increased CSF1 and CSF1R mRNA levels compared with the control shRNA group.

## Discussion

In this *in vitro* study, we demonstrate that S1PR2, a G-protein coupled plasma membrane receptor, plays an important role in regulating immune responses. Knockdown of S1PR2 by S1PR2 shRNA significantly reduced IL-1β, IL-6, and TNF-α release in BMMs stimulated by *A*. *actinomycetemcomitans*. Importantly, we are the first to demonstrate that S1PR2 is critical in regulating the differentiation of osteoclasts. Knockdown of S1PR2 suppressed osteoclastogenesis induced by RANKL. Mechanistically, we demonstrate that down-regulating S1PR2 decreased p-PI3K, p-ERK, p-JNK, p-p38, and p-NF-kBp65 protein expressions, and suppressed the mRNA levels of osteogenic factors including Nfatc1, Ctsk, Acp5, Oscar, Dcstamp, and Ocstamp.

Previous study showed that bacterial LPS and TNF-α enhanced both S1PR2 mRNA and protein expressions in human microvascular endothelial cells 24 h after the stimulations [29]. In our study, we observed a 65.2% reduction of S1PR2 mRNA level at 4 h after *A*. *actinomycetemcomitans* stimulation in BMMs without lentiviral treatment (Fig 1A). In contrast, in BMMs without lentiviral treatment, S1PR2 protein level increased 10.9% 1 h post bacterial stimulation and peaked (a 31.5% increase) at 2 h post bacterial stimulation compared with the basal level. At 4 h post bacterial stimulation, S1PR2 protein level fell to 19.0% above the basal level (Fig 2A and 2B). These data support the prior study’s [29] conclusion that bacterial LPS increased both S1PR2 mRNA and protein expressions. In our study, the mRNA level of S1PR2 might increase at early time points (before 4 h after bacterial stimulation) and decrease at 4 h after bacterial stimulation.

By using shRNA approach, we demonstrated that knockdown of S1PR2 in murine BMMs significantly attenuated the release of proinflammatory cytokines including IL-1β, IL-6, and TNF-α induced by bacterial stimulation (Fig 1). Our data are consistent with previous studies [17, 30], which demonstrated that S1PR2 knockout mice challenged with LPS exhibited significantly reduced serum IL-1β [17] and IL-6 [30] levels compared with the cytokine levels in wild type mice. Mechanistically, we demonstrated that knockdown of S1PR2 suppressed p-PI3K, p-ERK, p-JNK, p-p38, and p-NF-κBp65 protein expressions induced by *A*. *actinomycetemcomitans*. Previous studies showed that activation of PI3K, ERK, JNK, p38, and NF-κB signaling pathways contributed to the release of inflammatory cytokines induced by LPS. Human peripheral blood mononuclear cells (PBMCs) treated with a PI3K inhibitor (wortmannin) significantly inhibited LPS-induced chemokine C-X-C motif ligand 8 (CXCL8) and IL-6 release [31]. Raw 264.7 cells pre-treated with a NF-κB inhibitor (BAY 11–7082), an ERK inhibitor (U0126), a JNK inhibitor (SB600125), or a p38 inhibitor (SB203580) significantly suppressed TNF-α expression induced by LPS [32]. In addition, the ERK inhibitor (U0126) and the p38-MAPK inhibitor (SB 203580) also significantly inhibited IL-1β and TNF-α in PBMCs [33]. Since PI3K, ERK, JNK, p38, and NF-κB signaling pathways were up-regulated by bacterial stimulation (Fig 2) and activation of these signaling pathways contributed to the production of proinflammatory cytokines, suppressing these signaling pathways by S1PR2 shRNA might subsequently reduce IL-1β, IL-6 and TNF-α release induced by bacterial stimulation. Our previous study showed that treating murine BMMs with a multiple S1PRs modulator, FTY720 [24, 25], reduced IL-1β, IL-6, and TNF-α production induced by *A*. *actinomycetemcomitans* [26]. In this study, we further demonstrate that S1PR2 plays a critical role in modulating IL-1β, IL-6, and TNF-α production induced by bacterial stimulation.

Previously Gonda et al. [34] showed that Chinese hamster ovary (CHO) cells transfected with an AGR16/S1PR2 cDNA vector, significantly increased the generation of inositol phosphate and the activation of MAPKs (ERK, JNK, and p38) induced by S1P compared with parental CHO cells. In accordance with Gonda et al., our study demonstrated that down-regulating S1PR2 by specific S1PR2 shRNA suppressed PI3K and MAPKs (ERK, JNK, and p38) activities. In addition, our data supported that S1PR2 played a role in modulating NF-κB signaling. Among the Gi, Gq, and G_12/13_ proteins that couple with S1PR2, Gonda et al. [34] showed that treating CHO-AGR16/S1PR2 cells with a Gi protein inhibitor (pertussis toxin) inhibited S1P-induced inositol phosphates by 30% and p-ERK by 70%. In contrast, treating CHO-AGR16/S1PR2 cells with pertussis toxin has no significant effects on S1P-induced p-JNK and p-p38 activities [34]. These data suggest that PI3K and ERK signaling are regulated mainly via Gi protein, while JNK and p-38 signaling pathways are modulated mainly by pertussis toxin-insensitive proteins including Gq and G_12/13_ proteins.

S1P plays an essential role in modulating the migration of BMMs (osteoclast precursors) from blood to bone tissues and affects bone homeostasis [35]. Previous study showed that S1PR2 deficient mice exhibited moderate osteopetrosis compared with wild type mice [16]. Ishii et al. [16] explained that S1PR2 mediated a chemorepulsive role, which inhibited the migration of osteoclast precursors from blood to bone tissues and subsequently decreased osteoclastic bone resorption. In this study, we are the first to demonstrate that S1PR2 controls the differentiation of osteoclasts. Knockdown of S1PR2 by specific S1PR2 shRNA significantly reduced osteoclastogenesis induced by RANKL with or without *Aa*-media stimulation compared with controls.

Osteoclastogenesis involves fusion of osteoclast precursors to form multinucleated mature osteoclasts. It has been recognized that phosphoinositides are key signaling molecules that regulate osteoclastogenesis [27]. Activation of PI3K triggers Ca^2+^ release followed by activation of Nfatc1, a master transcription factor for osteoclastogenic gene regulation [9, 27]. In this study, BMMs treated with S1PR2 shRNA reduced p-PI3K before and after bacterial stimulation compared with controls (Fig 2). In contrast, BMMs treated with S1PR2 shRNA exhibited similar levels of p-ERK, p-JNK, p-p38, and p-NF-κB p65 before and 1 h after bacterial stimulation compared with these protein levels in cells treated with control shRNA (Fig 2). Since down-regulation of S1PR2 by S1PR2 shRNA suppressed osteoclastogenesis not only in cells stimulated with *Aa*-media but also in cells without *Aa*-media stimulation (Fig 3), our data support that PI3K signaling plays a major role in regulating osteoclastogenesis affected by S1PR2. Other signaling pathways, including ERK, JNK, p38, and NF-κB, might contribute to proinflammatory cytokine production, which subsequently promotes osteoclastogenesis. Our previous study showed that BMMs treated with FTY720 (a modulator of multiple S1PRs) inhibited p-PI3K expression, suppressed osteoclastogenesis, and reduced osteogenic factor Nfatc1, Ctsk, Acp5, and Oscar mRNA levels [26]. Although early studies supported that p-FTY720 bound with higher affinity with S1PR1, 3, 4 and 5 and served as a S1P agonist [36, 37], later studies demonstrated that p-FTY720 functioned as a noncompetitive inhibitor of multiple S1PRs including S1PR2 [24, 25]. In this study, we demonstrated that S1PR2 is critical in regulating osteoclastogenesis. Knockdown of S1PR2 by specific S1PR2 shRNA suppressed osteoclastogenesis and reduced the mRNA expressions of these osteoclastogenic factors. Moreover, we demonstrated that S1PR2 regulated the expressions of Dcstamp and Ocstamp induced by RANKL. Previous studies showed that Nfact1 also regulated Dcstamp and Ocstamp expressions [12, 13]. Thus, down-regulating Nfatc1 by S1PR2 suppressed these genes’ mRNA levels induced by RANKL.

In this study, the control shRNA also reduced bone resorption area (Fig 4) and decreased Nfatc1, Ctsk, Acp5, Oscar, Dcstamp, and Ocstamp mRNA levels (Fig 5) compared with cells uninfected with lentivirus before and after *Aa*-media stimulation. We speculate that the down-regulation of these osteogenic factors by the control shRNA might be caused by the non-specific effect of lentivirus. Since we observed reductions of RANK mRNA levels in cells treated with the control shRNA with or without *Aa*-media stimulation compared with RANK mRNA levels in cells uninfected with lentivirus, the down-regulation of RANK might subsequently cause reductions of Nfatc1, Ctsk, Acp5, Oscar, Dcstamp, and Ocstamp mRNA levels. Down-regulation of these osteoclastogenic genes can inhibit osteoclastogenesis and bone resorption. Additionally, in BMMs infected with a higher concentration of S1PR2 shRNA lentivirus (moi 50), the control shRNA also slightly reduced S1PR2 mRNA levels before and after bacterial stimulation compared with the S1PR2 levels in cells without lentiviral infection (Fig 1A). These findings suggest that excess lentivirus could cause a non-specific effect by down-regulating S1PR2 mRNA expression.

Bone marrow-derived mesenchymal stromal cells play a role in generation of RANKL, CSF1, and OPG, respectively. Previously, our study revealed that knockdown of S1PR2 by the S1PR2 shRNA in bone marrow-derived mesenchymal stromal cells had no significant effects on both RANKL mRNA and protein expressions compared with controls (data not shown). Although there was a trend of an increase of CSF1 mRNA in S1PR2 shRNA-treated cells compared with controls, there was no significant difference in CSF1 mRNA levels among these groups. We also observed a significant reduction of OPG mRNA levels in S1PR2 shRNA-treated mesenchymal stromal cells compared with controls (data not shown). These data were consistent with the results in Fig 5, which shows that total bone marrow cells treated with the S1PR2-shRNA had similar levels of RANKL and CSF1, but reduced level of OPG compared with cells treated with the control shRNA.

Constitutive S1P levels in tissues are very low (less than 1μM) because S1P can be degraded by S1P lyase or can be dephosphorylated by S1P phosphatase [1]. Our previous study [4] showed that treating BMMs with S1P (≤1μM) for 4 h did not significantly increase IL-1β, IL-6, and TNF-α mRNA levels compared with vehicle treatment. In addition, treating BMMs with both S1P (≤1μM) and *A*. *actinomycetemcomitans* for 4 h did not further enhance IL-1β, IL-6, and TNF-α mRNA levels compared with treating cells with *A*. *actinomycetemcomitans* alone [4]. Furthermore, BMMs from sphingosine kinase 1 deficient mice, which reduced S1P generation in response to bacterial stimulation, had no significant difference in IL-1β, IL-6, and TNF-α production induced by *A*. *actinomycetemcomitans* compared with wild type mice [4]. However, in this study, knockdown of S1PR2 by S1PR2 shRNA significantly reduced IL-1β, IL-6, and TNF-α protein levels induced by *A*. *actinomycetemcomitans* compared with controls. In consistent with our study, other studies [17, 30] also demonstrated that S1PR2 knockout mice challenged with LPS exhibited significantly reduced serum IL-1β [17] and IL-6 [30] levels compared with the cytokine levels in wild type mice. These studies suggest that S1PR2 might interact with toll-like receptors in regulating proinflammatory cytokine production induced by LPS. Besides S1P, other factors including bile acid [19], vitamin D [21], and insulin [22, 23] also interact with S1PR2, modulating cellular signaling pathways. Therefore, the effect of S1PR2 on proinflammatory cytokine production and osteoclastogenesis cannot be simply explained by modulating the S1P signal alone. Future studies need to determine how S1PR2 interacts with toll-like receptors, cytokine receptors, and other lipid receptors in modulating immune responses.

In conclusion, our study demonstrates that S1PR2 regulates proinflammatory cytokine production induced by bacterial stimulation and controls osteoclastogenesis. Our results suggest that suppressing S1PR2 might be a novel strategy to treat inflammatory bone loss diseases.

## Abbreviations

A. actinomycetemcomitans: Aggregatibacter actinomycetemcomitans
Acp5: acid phosphatase 5
BMMs: bone marrow-derived macrophages CFU, colony forming unit
Ctsk: cathepsin K
Dcstamp: dendritic cells specific transmembrane protein
EDG5: endothelial differentiation G-protein coupled receptor 5
ERK: extracellular signal-regulated kinase
GAPDH: glyceraldehyde 3-phosphate dehydrogenase
JNK: c-Jun N-terminal kinase
MAPKs: mitogen-activated protein kinases
M-CSF: macrophage colony-stimulating factor
NFATc1: nuclear factor of activated T-cells cytoplasmic calcineurin-dependent 1
NF-κB: nuclear factor-kappaB
Ocstamp: osteoclast stimulatory transmembrane protein
Oscar: osteoclast-associated receptor
OPG: osteoprotegerin
PI3K: phosphoinositide 3-kinase
RANKL: receptor activator of nuclear factor kappa-B ligand
S1P: sphingosine-1-phosphate
S1PR: sphingosine-1-phosphate receptor
TRAP: tartrate-resistant acid phosphatase
